# Fish oil-based lipid emulsion: current updates on a promising novel therapy for the management of parenteral nutrition-associated liver disease

**DOI:** 10.1093/gastro/gov011

**Published:** 2015-04-08

**Authors:** Shishira Bharadwaj, Tushar Gohel, Omer J. Deen, Robert DeChicco, Abdullah Shatnawei

**Affiliations:** Center for Human Nutrition, Department of Gastroenterology/Hepatology, The Cleveland Clinic Foundation, Cleveland, OH, USA

**Keywords:** intestinal failure, parenteral nutrition, parenteral nutrition-associated liver disease, fish oil-based lipid emulsion

## Abstract

Intestinal failure is characterized by loss of enteral function to absorb necessary nutrients and water to sustain life. Parenteral nutrition (PN) is a lifesaving therapeutic modality for patients with intestinal failure. Lifelong PN is also needed for patients who have short bowel syndrome due to extensive resection or a dysmotility disorder with malabsorption. However, prolonged PN is associated with short-term and long-term complications. Parenteral nutrition-associated liver disease (PNALD) is one of the long-term complications associated with the use of an intravenous lipid emulsion to prevent essential fatty acid deficiency in these patients. PNALD affects 30–60% of the adult population on long-term PN. Further, PNALD is one of the indications for isolated liver or combined liver and intestinal transplantation. There is no consensus on how to manage PNALD, but fish oil-based lipid emulsion (FOBLE) has been suggested to play an important role both in its prevention and reversal. There is significant improvement in liver function in those who received FOBLE as lipid supplement compared with those who received soy-based lipid emulsion. Studies have also demonstrated that FOBLE reverses hepatic steatosis and reduces markers of inflammation in patients on long-term PN. Future prospective studies with larger sample sizes are needed to further strengthen the positive role of FOBLE in PNALD.

## Introduction

Intestinal failure is defined as the inability of enterocytes to absorb necessary nutrients, water, electrolytes, vitamins and minerals either due to reduced length of the intestine or loss of its normal function [1]. Further, intestinal failure patients secondary to short bowel syndrome suffer from gastric acid hypersecretion, pancreatic enzymes inactivation, rapid bowel transit, gallstone formation, renal calculi and liver dysfunction [2]. Parenteral nutrition (PN) is the lifesaving treatment modality for providing necessary nutritional support to patients with intestinal failure [3]. Nearly 40 000 patients in the United States are currently on PN for survival [3]. The length of PN therapy can range from short term to long term depending on the patient’s medical and nutritional needs [4]. Short-term (2–6 weeks) indications include post-operative support for patients whose bowel function has not returned to normal or pre-operative support for malnourished patients [4]. Patients requiring long-term home PN (months to years or lifelong) are those with gastrointestinal dysmotility (e.g. scleroderma or gastroparesis) and short bowel syndrome due to extensive bowel resections [5]. However, PN is associated with a number of complications. Short-term complications include, but are not limited to, catheter-related sepsis and catheter-related mechanical complications. Long-term complications comprise liver failure and metabolic bone disease. Several strategies have been implemented to reduce short-term and long-term complications including catheter design, aseptic insertion technique, ethanol lock therapy, changes in PN composition and cyclic infusion of PN [6]. However, hepatobiliary dysfunction is an important reason for PN failure and one of the common indications for isolated liver or combined liver and intestinal transplantation [7]. The lipid component of PN has been thought to be involved in the pathogenesis of parenteral nutrition-associated liver disease (PNALD) and is presently considered an important modifiable risk factor in its prevention and treatment [8]. The aim of this review article is to illustrate the pathophysiological mechanism of PNALD and the promising role of fish oil-based lipid emulsion (FOBLE) in its prevention and possible treatment.

## Parenteral nutrition-associated liver disease

The incidence of PNALD in patients receiving long-term PN varies widely depending on the age group from 15–40% in adults and 40–60% in infants and neonates [8]. The prevalence of PNALD is greater in pediatric patients when compared with adults. The reasons for this are unknown [9]. Approximately 22% of deaths in patients on long-term PN are attributed to PNALD [10]. The clinical spectrum of hepatobiliary complications associated with PN range from steatosis, cholestasis, gallbladder sludge/stones, fibrosis and cirrhosis with significant overlap [11]. Patients with PNALD can present with either a single complication or a combination of complications [12]. Steatosis (defined as fat accumulation in the hepatocytes) can present as mild to moderate elevations in liver function tests and is usually benign. Cholestasis results from impaired secretion or obstruction of bile and is associated with elevations in alkaline phosphatase, gamma glutamyl transferase and conjugated bilirubin. Both steatosis and cholestasis are reversible with termination of PN. Bile stasis can also occur in these patients due to reduced cholecystokinin secretion caused by lack of enteral stimulation, decreased bile flow and gallbladder contractility [13]. The causes of PNALD are multifactorial and can be divided into nutrition-related causes and non-nutrition-related causes (Table 1). Non-nutrient reversible causes such as sepsis and hepatotoxic medications should be actively sought and treated first before attributing the cause of elevated liver function tests to PNALD [13].

**Table 1:** Parenteral nutrition and non-nutrient related factors associated with parenteral nutrition-associated liver disease

Parenteral nutrition-related factors	Non-nutrient related factors	
	Factors exclusive to pediatric population	Factors affecting both adult and pediatric population
Duration of parenteral nutrition	Prematurity	Recurrent septic episodes
High calorie intake	Low birth weight	Lack of enteral stimulation or delayed initiation of enteral feeding
Lipid infusions >1 g/kg/day	Duration on antibiotics after bowel surgery in infants	Length of remaining small bowel
Cysteine and taurine deficiency	Number of laparotomy surgeries in pediatric population	Underlying primary disease
Choline deficiency	Gastroschisis or jejunal atresia	Hepatotoxic medication
Manganese toxicity		

## Parenteral lipid emulsions: introduction and importance

Fats are esters of glycerol and fatty acids (FAs) [14]. FAs are hydrocarbon compounds with a methyl group at one end and a carboxyl group at the other end. FAs are usually classified broadly as saturated and unsaturated FAs based on the absence or presence of double bonds, respectively [14]. Unsaturated FAs are further classified depending on the number of double bonds into mono-unsaturated fatty acids having only one double bond and polyunsaturated fatty acids (PUFAs) having more than a single double bond [15]. Further, fats are condensed non-carbohydrate calorie sources for the human body. FAs are also the principal structural components of cell membranes. They act as second messengers and ligands for nuclear receptors [15]. Long-chain PUFAs are also precursors for eicosanoids and various other compounds such as resolvins and neuroprotectins [16, 17]. Although, the human body synthesizes most of the FAs, some should be supplemented through diet. These are called essential fatty acids (EFAs).

To avoid EFA deficiency, exogenous EFAs, which include linoleic (LA), linolenic (α-LA), and arachidonic acids (AA), must be provided [17]. Of these, only LA is required since α-LA has no known function in humans [18]. Further, AA can be synthesized if LA is provided. Platelet function, wound healing, immunocompetence, prostaglandin synthesis and integrity of the skin and hair all require EFAs [18]. Prevention of EFA deficiency in patients with intestinal failure is most commonly accomplished by administering intravenous fat emulsions (IVFEs) in patients with short bowel syndrome [19].

PN formulations before the advent of IVFE consisted mainly of glucose and amino acids [20]. Complications of high dextrose load in addition to deficiency of essential FA led to incorporation of lipid emulsions into PN. Hence, IVFE was introduced in the 1960s to both prevent EFA deficiency and serve as a major source of non-protein calories [21]. Further, IVFE minimizes respiratory and metabolic stress and prevents fatty infiltration of the liver. Lipid (9 kcal/g) emulsions also provide more energy-rich source compared with glucose (3.4 kcal/g) and amino acids (4 kcal/g) [21]. The PN volume is reduced with the use of IVFE, which may be helpful for patients with fluid overload [22]. Although the time of development of EFA deficiency is variable and depends upon the patient’s nutritional status, nature of disease and age, biochemical EFA deficiency develops after 4 weeks of fat-free PN in hospitalized patients; patients on home PN, however, may not develop it for months after receiving fat-free PN [23]. The cause for this discrepancy is unknown. It has also been reported that neonates develop biochemical signs of EFA deficiency as early as the second day of life and within two weeks of fat-free PN [24]. It is presently advised to start IVFE within seven days of starting PN to prevent EFA deficiency [23]. Daily requirements for EFA are unknown; however, studies have shown that EFA deficiency can be prevented by providing a minimum of 2–4% of total caloric intake as LA and 0.25–0.5% as α-LA [23].

The introduction of a successful IVFE in 1961 was one of the major advances in the administration of PN [25]. A cotton seed oil emulsion was the first IVFE (Lipomule) to be available in the United States, but it was immediately withdrawn from use due to adverse reactions including chills, fever, nausea, dyspnea, hypoxia, hypotension and fat embolism [25]. In the early 1960s, a soybean oil-based IVFE (Intralipid) was introduced and was well tolerated [26]. A study from Sweden reported that only eight patients developed a significant adverse reaction to an IVFE out of the 1.6 million units infused. Of those eight patients, only one adverse reaction was attributed to Intralipid infusion [26]. These results demonstrated the safety of soybean oil-based IVFE, which offered a source of EFA as well as an alternate calorie source to dextrose since complications of hyperosmolar glucose solutions, including liver steatosis, were increasingly recognized along with manifestations of fat-soluble vitamin deficiencies [27].

## Soybean oil-based lipid emulsion: its role in pnald

Historically, the IVFE was first derived from plant/vegetable oils because they are rich in EFAs [27]. The commercially available soybean oil-based lipid emulsion (SOBLE) products in the United States are intralipid-soybean oil emulsions based on, or a combination of, soybean and safflower oils (Liposyn) [27]. These contain LA and α-LA approximately in the proportion of 5.5:1 along with high content of phytosterols and low levels of α tocopherol [28]. Both LA and α-LA have similar metabolisms with the latter being the preferred substrate for the enzymes delta-6-desaturase, elongase and delta-5-desaturase [29]. AA is derived from LA, while eicosapentaenoic acid (EPA) and docosahexaenoic acid (DHA) are derived from α-LA [29]. Further, AA competes with EPA and DHA for incorporation into cell membranes and its further metabolic pathway [30]. Eicosanoids are important end products of all these fatty acids: AA, EPA and DHA. AA forms 2-series prostaglandins, thromboxanes and 4-series leukotrienes [31]. These compounds have pro-inflammatory properties. On the other hand, the products of metabolism of EPA and DHA (i.e. 3-series prostaglandins, thromboxanes and 5-series leukotrienes) are shown to have anti-inflammatory properties [32].

The high content of n-6 PUFA in SOBLE impacts the immune status of patients by affecting cellular immunity and increasing cytokine production by activating nuclear factor-kB pathway [33]. Cytokines, namely interleukin-1 (IL-1), IL-6 and tumor necrosis factor alpha (TNF-α) lead to disruption of hepatobiliary transport systems and thus result in cholestasis [33]. A demonstration by Beckh *et al.* showed reduction of bile flow and bile acid secretion in rat liver after perfusion of prostaglandin F2α, D2 and E2, adding further support to the role of n-6 PUFA in PNALD [34]. SOBLE has high amounts of n-6 PUFA (approximately 85%), which accentuates lipid peroxidation andthereby depletes serum levels of tocopherols (an anti-oxidant that further aggravates inflammation) [35]. Animal model studies on piglets and rat hepatocytes have identified phytosterols as having an inhibitory effect on bile acid secretion and excretion [36]. Further, beta-sitosterol, the most abundant phytosterol in SOBLE, is known to have inhibitory effects on farnesoid X receptor (FXR), a primary bile acid sensor and the key molecule in bile acid hemostasis [36]. Also, FXR regulates bile acid homeostasis by its action on cholesterol 7 alpha hydroxylase, the rate-limiting enzyme in bile acid synthesis [37]. Further, FXR stimulates the transcription of the gut enterokine fibroblast growth factor 19, which mediates the repression of cholesterol 7 alpha-hydroxylase [37]. All of these mechanisms are involved in the pathogenesis of hepatobiliary dysfunction in patients with PNALD [38]. With the increasingly recognizable role of SOBLE in PNALD and other nutrition-related complications, IVFEs have evolved through generations by replacing n-6 PUFA with medium chain triglycerides (MCTs) or n-3 PUFA [38].

## Fish oil-based lipid emulsion: beneficial role in pnald

Fish oil, as a rich source of n-3 PUFA with lower phytosterol and higher alpha tocopherol content, is gaining popularity as an alternative source of IVFE in PN, either alone or in combination with others [39]. Further, FOBLE contains high concentrations of EPA and DHA, which are known to have anti-inflammatory properties [39]. They compete with AA and change the composition of cell membranes [40]. Additionally, hydrolyzed products of these membrane phospholipids modulate eicosanoid and cytokine biology [41]. Furthermore, they have a suppressive effect on cellular immunity including lymphocytes, neutrophil chemotaxis and T cells along with antigen presentation [41]. Hence, n-3 based IVFEs are finding their way as a combined nutritional and pharmacological agent for use in pro-inflammatory states such as sepsis and acute respiratory distress syndrome.

The beneficial effect of n-3 PUFA in inflammatory states has been demonstrated in a number of studies [42]. Liang *et al.* reported that colorectal cancer patients who received a mixed emulsion of FOBLE and SOBLE as a part of PN post-operatively for 7 days were found to have a significant decrease in serum IL-6 levels and increase in the CD4+ / CD8+ ratio [42]. Similarly, another study reported decreased levels of IL-1, IL-6, IL-10 and TNF-α in their patients who received n-3 PUFA-enriched PN post-operatively compared with those who received an isocaloric MCT/LCT (long-chain triglyceride) enriched PN [43]. Furthermore, Weiss *et al.* reported that perioperative administration of n-3 PUFA downregulated the inflammatory response favoring better outcomes in surgical trauma patients [44].

FOBLE has gained popularity in recent years for patients on PN with liver dysfunction, not only as a supplement for SOBLE for prevention of PNALD but also as a therapeutic modality for reversal of PNALD [45]. The underlying mechanism for reversing liver dysfunction is not clearly known. Pscheidl *et al.* demonstrated in two distinct rat model studies an improved intestinal, portal perfusion and enhanced bactericidal defense of splanchnic circulation with FOBLE [46]. The lower pro-inflammatory properties of n-3 FA and decreased amounts of phytosterols in FOBLE compared with SOBLE are thought to play a role in the hepatoprotective nature of these emulsions [47].

Initially, the evidence for n-3 FA enriched IVFE reversing steatosis and cholestasis was demonstrated in animal model studies [48, 49]. Recently, studies have investigated the beneficial effect of FOBLE in pediatric and adult populations [50, 51]. One of the earlier studies conducted in two neonates with short bowel syndrome on PN reported reversal of liver disease after supplementation of n-3 PUFA [50]. Further, recent studies involving adult surgical and critically ill patients have demonstrated favorable response. A study involving patients who underwent major abdominal surgery reported improved liver functions in those who received FOBLE compared with those who received SOBLE [52]. Another study of gastrointestinal surgical patients receiving FOBLE in PN post-operatively reported improved liver functions compared with those receiving MCT/LCT combination [53]. Further, Mertes *et al.* found better liver tolerance in surgical and intensive care unit patients receiving a combination emulsion, SMOF (soybean oil, medium chain triglycerides, olive oil and fish oil) compared with those receiving SOBLE alone [54]. Additionally, one study of IF patients on PN for four weeks reported lower levels of liver enzymes in patients receiving SMOF compared with those receiving SOBLE [55]. Furthermore, a recent case report of an adult with PNALD by Burnes *et al.* showed reversal of hepatic dysfunction with change of IVFE from soybean to FOBLE [56]. Jurewitch *et al.* demonstrated in their case report that n-3 enriched lipid emulsion (100% fish oil) normalized PN-induced cholestasis and resolved histochemical and ultrastructural abnormalities in an adult patient [57]. Concerns about the use of FOBLE monotherapy include the development of EFA deficiency and bleeding. One report described burr cell anemia in an infant receiving FOBLE monotherapy, which resolved with its discontinuation [58].

In summary, PNALD is a common complication of long-term PN that is in part due to the type and amount of IVFE. FOBLE has a lower amount of phytosterols and higher concentration of n-3 FA compared with SOBLE, which may prevent or potentially reverse PNALD. FOBLE may also have less pro-inflammatory effects, less immunosuppression and more antioxidant effects compared with standard SOBLE. Prolonged bleeding time, though rare, could be a drawback of fish oil use. Also, the currently available data on the clinical benefits of fish oil are based on a diverse group of patients. Future studies are needed to further strengthen the results of the promising role of FOBLE for prevention and treatment of PNALD in adults receiving long-term PN.

*Conflict of interest statement:* none declared.
